# Demographic insights into paternal genetic diversity and regional substructure in the Spanish Roma

**DOI:** 10.1186/s12864-025-12210-8

**Published:** 2025-11-07

**Authors:** Giacomo Francesco Ena, Aaron Giménez, Annabel Carballo-Mesa, Marcos Araújo Castro e Silva, David Comas

**Affiliations:** 1 Institut de Biologia Evolutiva (CSIC-UPF), Departament de Medicina i Ciències de la Vida, Universitat Pompeu Fabra, Barcelona, Spain; 2 Facultat de Sociologia, Universitat Autònoma de Barcelona, Barcelona, Spain; 3 Facultat de Geografia i Història, Universitat de Barcelona, Barcelona, Spain

**Keywords:** Iberian roma, Population genetics, Migration, Demography, Y-chromosome

## Abstract

**Background:**

The Iberian (Calé) Roma constitute one of the largest Roma communities in Europe, yet their internal genetic structure and connections to other Roma groups remain understudied. This study explores the microgeographical structure of the Iberian Roma and their relationships with other Roma groups by analysing paternal lineages using 17 Y-chromosome short tandem repeat markers in a geographically stratified sample of 173 Spanish Roma individuals.

**Results:**

The haplogroup distribution patterns indicate that the paternal genetic profile of the Spanish Roma is shaped by founder effects, population bottlenecks, and multiple admixture events with non-Roma groups. Haplogroups H and J2a1b dominate the genetic landscape, reflecting their South Asian origin and subsequent dispersal patterns through West Asia into Europe. A distinctive feature of the Spanish Roma is the high frequency of haplogroup R1b, indicating significant gene flow from non-Roma Iberian populations. The absence of North African or Jewish genetic influences rules out the possibility of a North African migration route for the Calé Roma into the Iberian Peninsula. Microgeographical analyses (AMOVA) reveal substantial genetic substructure among Calé Roma across Spanish regions, consistent with historical isolation and localised gene flow. Additionally, a striking sex-biased admixture is observed when comparing the current results with previous mitochondrial DNA (mtDNA) data, with paternal South Asian ancestry being twice as high as maternal contributions, suggesting that Roma communities have historically been more inclined to integrate non-Roma women.

**Conclusions:**

The genetic landscape of the Iberian Roma is shaped by a complex history of founder effects, admixture, and isolation. The observed genetic substructure and sex-biased admixture reflect historical social dynamics. These results contribute to the broader understanding of Roma genetic diversity and demography in Spain and underscore the importance of integrating Y chromosome, autosomal, and mtDNA data in future studies.

**Supplementary Information:**

The online version contains supplementary material available at 10.1186/s12864-025-12210-8.

## Introduction

The Romani are the largest transnational ethnic minority in Europe. Although traditionally nomadic or semi-nomadic, most of them have been settled for centuries and generally share a common identity and cultural traditions [1–3]. The history of Roma has been reconstructed through linguistics, historical records, and anthropological studies, which combined with genetic research, traced their origins in the northwestern region of India and southeastern Pakistan [4–8]. The Roma have a complex social structure that transcends the concept of national identities, characterised by a tradition of living in relatively closed social groups [1, 3], and using distinct varieties of the Romani language alongside local languages of the countries in which they reside [5, 9]. Romani language is classified within the Indo-Aryan branch of the Indo-European language family, and its dialects—despite regional variation—share a common origin [9, 10]. Linguistic influences acquired during the diaspora—such as Farsi loanwords from early stages of migration and Greek loanwords from the Byzantine period—reflect key historical contact zones [11, 12]. As such, linguistic research has provided an independent line of evidence for tracing Roma migrations, supporting their origins in Northern India [5, 13].

The Roma community is widespread across Europe, with one of the largest groups, the Calé Romani in Iberia (Spain and Portugal), where some members still speak the endangered Caló language [14, 15]. Historical records indicate that the Roma arrived in the various kingdoms of what is now Spain in 1425 [16] and in Portugal likely earlier than 1521 [15], the year of their first documented mention, having migrated from Eastern and Central Europe [16, 17]. This marks six centuries of Roma presence in Spain, a history that has contributed to the country’s cultural and social development. While popular myths and some scholars have hypothesised that the Iberian Roma may have migrated from North Africa [4, 18], or share close genetic connections with Jewish groups [19, 20], previous studies have found no genetic evidence to support these claims [21, 22]. In the fragmented political landscape of the time, they sought safe conducts by presenting themselves as pilgrims traveling to Santiago de Compostela, which most rulers initially granted [23]. The arrival of the Roma in present-day Spain coincided with a period of social and political turmoil, characterised by the expulsion of Jews and Muslims by the Christian kingdoms. Although initially tolerated, the Roma soon encountered increasing restrictions and persecution, with policies aimed at their forced settlement or expulsion [17, 23, 24]. In the 19th and 20th centuries, internal migrations further displaced Roma populations within Spain, potentially increasing admixture among groups [23]. Despite their centuries-long presence in Europe and some recent improvements in their conditions, the Roma have historically faced varying degrees of social marginalisation, both from the broader population and through systemic discrimination [24–26].

Over the past 25 years, genetic research on Spanish Roma has often focused on medical issues [27–30]. However, several studies on uniparental DNA have included Spanish Roma individuals, with two specifically examining Spanish mtDNA [31, 32], collectively revealing evidence of bottlenecks, identifying founder lineages, and uncovering sex-biased admixture patterns [33–36]. In addition, two studies focusing on Roma autosomal data have also included Spanish Roma samples [7, 37], while the most recent and comprehensive research to date consists of an in-depth whole-genome array study on Iberian Roma [21]. These studies provide extensive insights into their genetic diversity, relationships with other populations, population history, and the impact of socio-cultural practices on genetic variation and population structure. However, Y-chromosome genetic diversity remains largely understudied, with only one study examining paternal lineages in Portugal [22], and another including a limited sample size of Spanish Roma that did not specifically focus on this group [33], and none dedicated solely to the Spanish Roma. This leaves a critical gap in understanding the paternal genetic history of the Spanish Roma, which is essential for a more comprehensive view of their genetic diversity, admixture patterns, and historical migration. Y-DNA studies, in particular, offer unique insights by revealing sex-biased gene flow and the presence of founding lineages—elements not captured by autosomal DNA analysis. These features are crucial for understanding male-mediated gene flow and its impact on the Roma’s genetic profile and demographic history.

To address these gaps, this community-driven initiative conducted in collaboration with FAGiC (Federation of Roma Associations of Catalonia), analysed up to 27 Y-DNA Short Tandem Repeats (STRs) data from 173 Spanish Roma volunteers, including 133 newly genotyped individuals. The primary aims of this study were to: (i) evaluate Spanish Roma Y-chromosome diversity and whether their patrilineal lineages are geographically substructured; (ii) evaluate the paternal genetic relatedness with other Roma and non-Roma populations from Europe, investigating potential influences from North Africa and Jewish groups; (iii) infer the population history of the Roma patrilineal lineages through Europe and the Iberian Peninsula. This comprehensive study provides a detailed picture of the patrilineal genetic diversity of the Iberian Roma, offering insights from both micro-geographical and broader perspectives on European Roma history and demography.

## Materials and methods

### Samples

We genotyped Y-STRs from 133 Spanish Roma volunteers using saliva samples. The collection of the samples was conducted under the umbrella of the ‘El Camí del Poble Gitano: una història de diversitat’ project [38], in collaboration with the Roma FAGiC association (*Federació d’Associacions Gitanes de Catalunya*). Participants were selected randomly and based on their self-identified Roma ancestry, with recruitment facilitated by FAGiC, which helped identify volunteers from the Spanish Roma community. To maximise sample inclusion, we used two different Y-STR kits: the Yfiler^®^ Plus PCR Amplification Kit (27 markers) and the AmpFlSTR^®^ Yfiler^®^ PCR amplification kit (17 markers), based on the availability of samples and kits at the time of genotyping.

### Y-STR genotyping

A total of 64 Spanish Roma samples were typed for the 27 Y-STR loci included in the Yfiler^®^ Plus PCR Amplification Kit (Applied Biosystems/Thermo Fisher Scientific) and additional 69 Spanish Roma samples were typed for the 17 Y-STR loci included in the AmpF*l*STR^®^ Yfiler^®^ PCR amplification kit (Applied Biosystems, Inc.). Resulting amplicons were separated on an ABI 3730 XL Genetic Analyzer using ABI GeneScan 600 LIZ as an internal size standard, and fragment lengths were estimated by GeneMapper v4.1 [39]. Y-STR alleles were assigned by comparison with an allelic ladder provided by the manufacturer. Allelic nomenclature follows the recommendations of the International Society for Forensic Genetics (ISFG) [40].

After genotyping, 133 Spanish Roma samples were included in the dataset, while the inclusion of 40 Spanish Roma samples from Martinez-Cruz et al. [33] brings the final number to 173. For regional-scale analyses, the Spanish Roma and non-Roma populations (references in Table S1) were grouped into five Iberian geographical regions (Centre, North, South, West, and East) based on their sampling location, following the approach used in Aizpurua-Iraola et al. [32] and Ena et al. [21] (sample distribution in Figure S1).

A total of 50 reference populations (10,307 individuals; Table S1) with Y-STR frequency data were included for comparisons with the Spanish Roma population, 11 of which are Roma groups from different European countries. All collections, biogeographical origins, reference publications, and total number of individuals analysed, along with detailed citations for each reference dataset obtained from supplementary materials of previously published studies, are listed in Supplementary Table S1. These datasets do not have centralised accession numbers but are publicly accessible via the cited sources.

To ensure compatibility with a broader range of reference populations, analyses were conducted using only the 17 Y-STR markers included in the AmpF*l*STR^®^ Yfiler^®^ PCR amplification kit. Y-chromosomal haplogroup prediction was primarily conducted using Whit Athey’s Haplogroup Predictor v5 (27-Haplogroups version) based on allele frequencies from 17 Y-STR loci. However, where additional Y-STR loci were available, predictions incorporated these additional markers to enhance accuracy. Whit Athey’s Haplogroup Predictor is based on the Bayesian-allele-frequency algorithm [41]; for our analysis we set the fitness score to 0, the Bayesian probability to 85%, and applied equal priors. In the case of intermediate alleles, repeat numbers were rounded to the nearest integer; missing alleles were coded as ‘99’ in input files and considered as missing data, as performed in previous studies [42]. For the prediction, DYS389II is represented as the sum of the two parts of this marker.

### Statistical analysis

Haplotype diversity (HD) of the Spanish Roma population samples was assessed using Nei’s HD formula [43] and calculated with R software [44]. Haplotype frequencies were determined by direct counting. We then calculated the confidence interval using the bootstrap method with 10,000 iterations, implemented via the ‘boot’ package in R [45]. The genotypic data for Y-STR in 133 novel individuals are presented in Supplementary Table S2. A permutation test was performed to compare the number of distinct haplogroups in Spanish Roma with those in other Roma populations, based on 10,000 permutations.

Population pairwise genetic distances (Slatkin R_ST_) [46] were calculated using Arlequin version 3.5.1.2 [47], after converting raw data to the arp format via a custom script. The analysis used 17-locus haplotypes from individuals belonging to haplogroups H, J2a1b, R1b, and I2a(x) (defined as the grouping of I2a, I2a(xI2a1), and I2a1). The statistical significance of the R_ST_ values generated, based on a stepwise mutation model, was ascertained through permutation tests (10,000 iterations). The migration rate (M) matrix was computed for all the Spanish individuals using Arlequin. A multidimensional scaling (MDS) analysis was performed using the metaMDS function from the vegan package [48], and plots were created using ‘ggplot2’ to visualise the genetic distances among the populations examined, based on the R_ST_ pairwise matrix. The patterns of genetic differentiation were further assessed through an analysis of molecular variance (AMOVA) conducted in Arlequin.

### Median-joining networks

Y-STR haplotypes of individuals belonging to the J2a1b, H, R1b and I2a(x) haplogroups were used to generate Median-Joining networks using NETWORK 10.2.0.0 (www.fluxus-engineering.com). The networks were generated using the median-joining algorithm, with the weight of each STR locus assigned a value from 1 to 10, inversely proportional to the STR variance, following references [49, 50]. The Maximum Parsimony (MP) option was employed to infer the simplest topology with a good fit to the data. In the case of intermediate alleles, repeat numbers were rounded to the nearest integer, following the approach of previous studies [51]. For calculating networks, we excluded the constitutively duplicated loci (385a/b), as indicated by the Network User Guide, while we retained DYS389I/II after subtracting the number of repetitions in DYS389I from DYS389II. Any missing data or deleted alleles were replaced with the standard code ‘99’ in the input files. To enhance interpretability of the analysis and address computational challenges caused by the large number of samples, we applied random sampling to limit the reference sample size to 20 in H, R1b and I2a(x) networks.

### Time estimates

Y-STR haplotypes were used to estimate the time to the most recent common ancestor (TMRCA) of the H haplogroup, along with the J2a1b, R1b, and I2a(x) sub-haplogroups prevalent among Spanish Roma. To achieve this, the rho statistic (*ρ*) and weighted rho (*ρW*) were computed using a modified version of the weighted rho method [52], adjusting the mutation rates to fit our input data. We first applied the pedigree mutation rates for each STR obtained from the Y-Chromosome STR Haplotype Database (YHRD, www.yhrd.org). In addition, we applied the rho method using a median pedigree-based mutation rate of 2.5 × 10^−3^, as described by Goedbloed et al. [53] and implemented by Pamjav et al. [54]. The DYS385 marker was excluded from the calculations. The statistical significance of differences in time estimates was evaluated by comparing the standard deviation of the dates.

### Migration rates in the Roma population

We used MIGRATE version 5.0.6 [55], a software based on coalescent theory that applies Bayesian inference to jointly estimate all parameters of a demographic model, to infer migration patterns in the Roma population from Spain and other relevant Roma populations across Europe. The following parameters were found to provide the models with the highest likelihood after a series of exploratory analysis: one single long chain was run in three independent replicates with a sampling increment of 500 and 2,000 recorded steps, while the number of discarded trees per chain (burn-in) was set to 2,500. Based on the increment value and the number of discarded trees, each sample was visited 3,000,000 times. All models were inferred with uniform priors, using two distinct prior settings: one for effective population size and migration (Min: 0.0, Max: 50.0, Delta: 2.0), and another for divergence and divergence standard deviation (Min: 0.0, Max: 50.0, Delta: 5.0).

Metropolis-Coupled MCMC (“MCMCMC”) or “heating” was applied for auxiliary searches with more permissive acceptance criteria. The search was executed with four chains at different temperatures (1.0, 1.5, 3.0, and 10,000) with an adaptive heating scheme that manipulated the temperatures according to their swapping success, as described in previous studies [51, 56, 57].

Gene flow was explored at two geographic scales: investigating the dispersal patterns within the Iberian Peninsula, and examining long-distance movements from the Balkans to Western Europe. First, we inferred the migration rate between Iberian regions, considering the Spanish Roma divided by geographic regions, also including the reference Portuguese Roma. We employed five distinct population history models to investigate migration patterns, following the approach of Almohammed et al. [51]: (i) the first model assumed all populations belonged to the same panmictic population; (ii) the second model entailed unidirectional gene flow from one population to another (East to West); (iii) the third model accounted for divergence from a common ancestral population; (iv) while the fourth model incorporated both divergence from the ancestral population and ongoing immigration; (v) the fifth model included both divergence from the ancestral population and ongoing immigration in two directions (East to West and *viceversa)* (Figure S2). Subsequently, we conducted the analysis on a continental scale, where pairwise comparisons were made between Spanish Roma and Roma from three other European countries: Greece, Romania and Slovakia. For this analysis, we used the same previously described migration models.

To assess the relative strengths of the model fits, log marginal likelihoods were used to calculate Bayes factors using the script provided by the developer. The magnitudes of the Bayes factors provided evidence for the degree of dissimilarity between the models, which informed us about the relative fit of each model to the data.

## Results

### Y-chromosome haplogroup composition and diversity of Spanish Roma

**Fig. 1:**
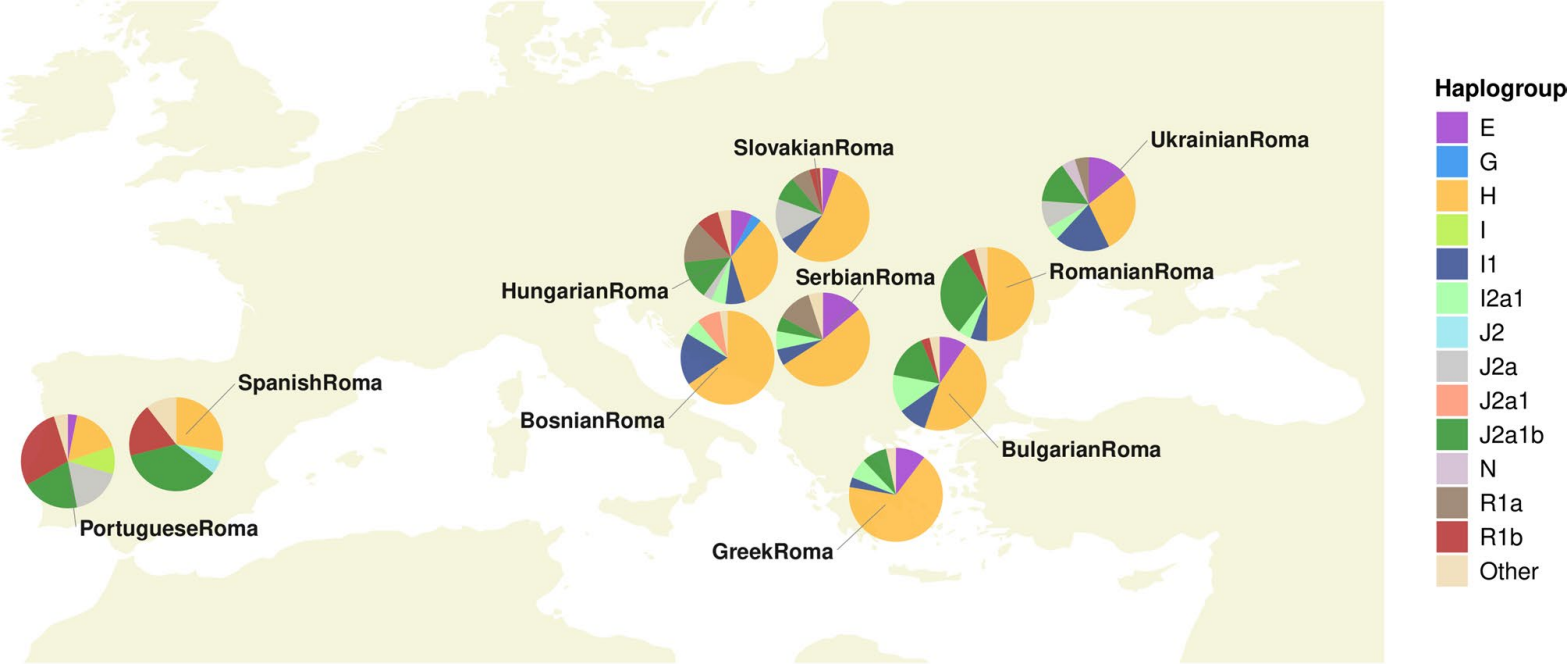
Geographic distribution of Y-chromosome haplogroups in European Roma populations. Pie charts show the frequencies of the inferred haplogroups in each population. Haplogroups with frequencies below 3% are labelled as “Other” in the legend

The largest proportion of Y-chromosome lineages in Spanish Roma (35.8%) are assigned to haplogroup J2a1b, followed by H (27.7%), R1b (18.5%), J2 (3.5%), and I2a1 (3.5%), with smaller percentages (< 3%) for the remaining haplogroups (Table S3). Differences are observed between Spanish Roma and other Roma groups (Table S4), with the exception of Portuguese Roma, who display a haplogroup frequency profile similar to that of Spanish Roma. Spanish Roma exhibit notably lower frequencies of the South Asian haplogroup H and higher frequencies of haplogroups R1b and J2a1b, both associated with West Eurasian regions (*i.e.*, Europe and West Asia), compared to most Central and Eastern European Roma populations (Fig. 1). These findings suggest higher levels of gene flow between Iberian Roma (Spanish and Portuguese Roma) and non-Roma Western European populations, in contrast to other European Roma groups. Among the haplogroups identifiable using Athey’s method, no significant differences in the number of distinct haplogroups were observed between the Spanish Roma and other Roma populations (permutation test *p*-value = 0.0985).

To investigate potential admixture from North African and Jewish populations, which may have occurred during the period of cohabitation in the Iberian Peninsula in the 15th century, we examined the presence of Y-DNA haplogroups linked to these ancestries in the Spanish Roma. Our analysis revealed no significant North African influence on the Y-DNA composition of the Spanish Roma, as evidenced by the complete absence of native North African E haplogroups, such as E-M81 (which represents around 40–46% in North Africa) and the internal clade E-V65 of E-M78 (found in around 3%) [58, 59]. Similarly, we found no genetic connection between Spanish Roma and Jewish populations, as markers typically associated with Jewish ancestry (such as J1-P58, J2-M172, E-M34, and R1a-M582) [60–63] were absent in Spanish Roma. Having ruled out significant admixture and shared ancestry from North African and Jewish populations, we next turned our attention to the internal diversity of haplogroups within the Spanish Roma.

**Fig. 2:**
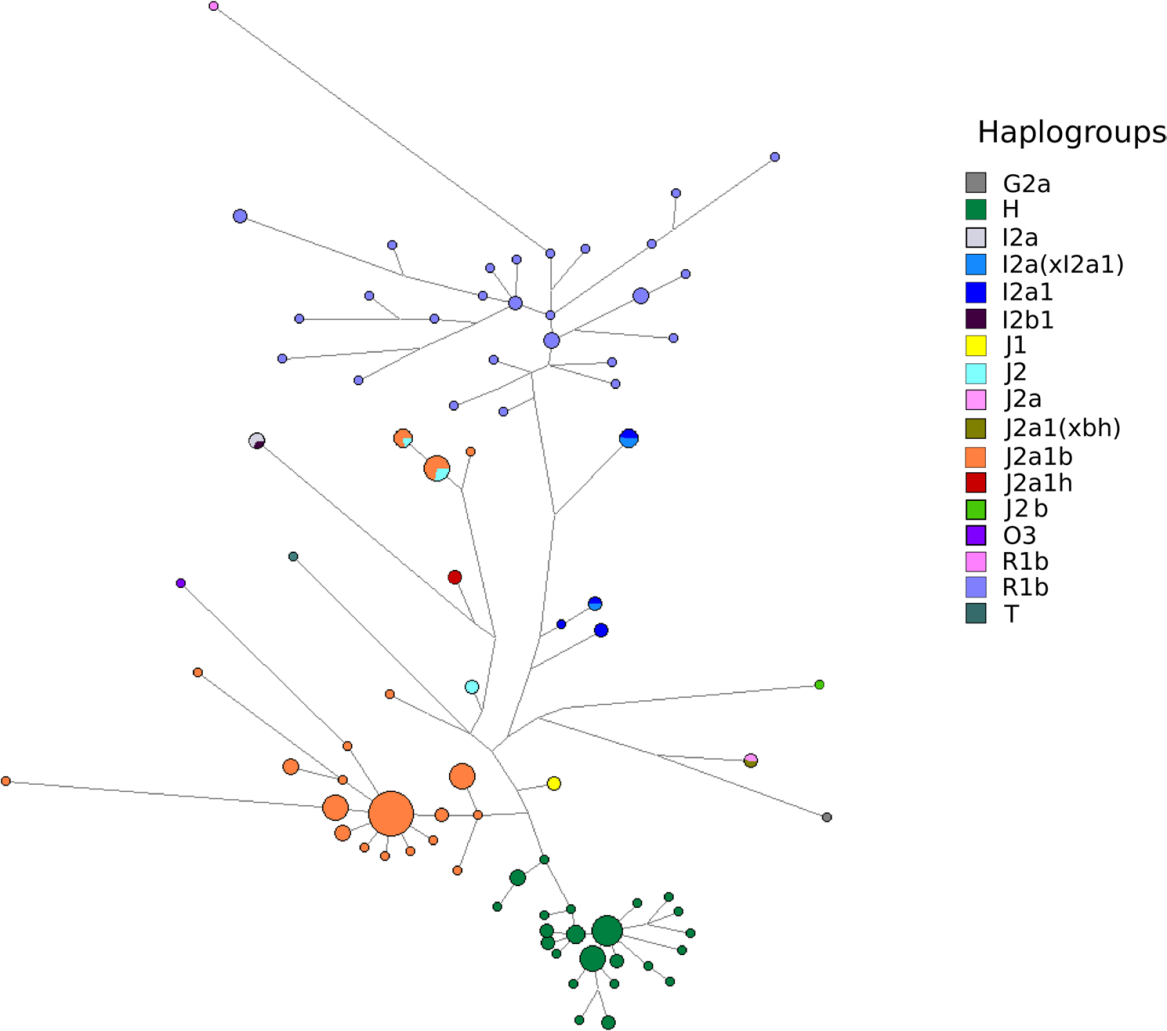
Median-joining network of 173 Spanish Roma individuals based on 17 Y-chromosome STR markers. Colours represent the inferred haplogroups derived from the STR profiles

To explore this further, we constructed median-joining networks based on the Y-STR haplotypes (15 loci) for all Spanish Roma individuals, as well as independent networks for each of the four most frequent haplogroups (H, J2a1b, I2a(x), and R1b). Haplogroups were assigned within the network based on predicted clusters and, where available, Y-SNP data from reference populations. The resulting network analysis revealed distinct patterns of haplogroup diversity, highlighting the dominant features of each lineage. The dominant feature of the general Spanish Roma network (including all haplogroups; Fig. 2) is the tight clustering of all H haplotypes within short branches, indicating minimal diversity. Similarly, J2a1b exhibits low diversity, although with some haplotypes more distantly located from the main cluster. R1b individuals are more dispersed across the network, reflecting greater diversity, while the other haplogroups are distributed between the main clusters without distinct patterns, except for a small subgroup of I2a(x). This network structure suggests that the H and J2a1b haplogroups in the Spanish Roma population may have experienced a founder effect, where a small initial group of ancestors contributed to the genetic pool, resulting in reduced diversity within these haplogroups compared to others.

Building upon the general network analysis of Spanish Roma, we next examined the diversity of individual haplogroups across all populations in our study. Y-STR median-joining networks for the founder haplogroups H and J2a1b show no clear clustering or differentiation within the Spanish Roma and exhibit limited diversity (Figures S3, S4, S5), suggesting an early and common divergence from other Roma groups. In contrast, the R1b and I2a(x) networks display considerable genetic diversity and a lack of clear structure among the Roma, indicating that the diversity in these haplogroups likely arose from independent admixture with local European populations during the Roma’s migration through Europe (Figure S6 and Figure S7). Overall, these findings suggest that the Spanish Roma carried relatively low diversity for the founder haplogroups H and J2a1b upon their arrival in Spain, but incorporated a broader range of R1b and I2a(x) sub-haplogroups through admixture with local populations during their diaspora to the Iberian Peninsula.

**Fig. 3:**
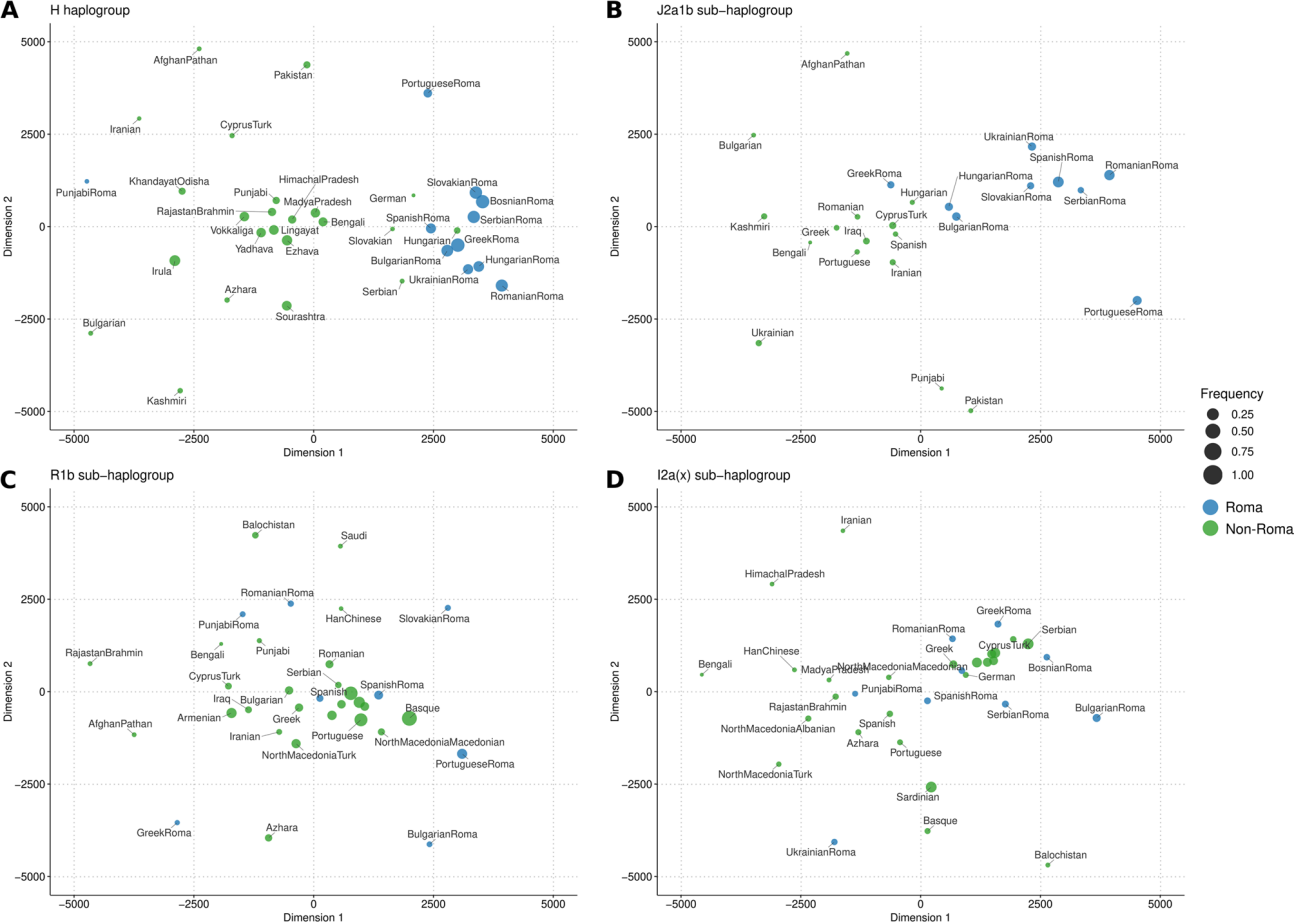
Multidimensional scaling (MDS) plots of the four major haplogroups found in Spanish Roma, based on Rst distances: (**a**) Haplogroup H; (**b**) Haplogroup J2a1b; (**c**) Haplogroup R1b; (**d**) Haplogroup I2a(x). Roma populations are shown in blue and non-Roma populations in green. The size of the circles represents the population frequency of the haplogroups

To further investigate genetic differentiation within these haplogroups, we performed an MDS analysis based on pairwise genetic distances (Slatkin R_st_). The MDS results for the H and J2a1b haplogroups (Fig. 3a-b) show Roma populations tightly clustered, with a higher frequency of these haplogroups compared to non-Roma groups, highlighting the effects of founder events and genetic drift after their arrival in Europe. In contrast, for R1b and I2a(x) (Fig. 3c-d), show a more dispersed distribution among Roma populations, with no distinctive clustering and lower frequency relative to non-Roma groups, suggesting greater diversity from multiple introgressions and a more recent introduction into the Roma gene pool after their arrival in Europe.

To explore the timing of these genetic events, we estimated the time to the most recent common ancestor (TMRCA) for the four major haplogroups across three contexts: the entire dataset, all Roma populations, and Spanish Roma (Figure S8 and Table S5). In all cases, the TMRCA dates were more recent for the Spanish Roma, reflecting their more recent differentiation. The inferred dates predate the suggested out-of-India diaspora, which occurred over a thousand years ago [2, 4, 6, 7], hinting that the internal haplogroup diversity in the Roma gene pool predates their migration into Europe. Furthermore, this implies that the diversity observed in the R1b, J2a1b, and I2a(x) haplogroups was already present at the time of introgression, with no significant diversification detected subsequently within the Spanish Roma. These findings indicate that the Y-chromosome diversity in Roma groups reflects a series of distinct ancestry sources: (1) early genetic diversity from their South Asian ancestors, particularly for haplogroup H, which was present in South India; (2) the introduction of haplogroups such as J2a1b during their migration out of South Asia, but before reaching Europe; and (3) further admixture events in Europe involving haplogroups such as I2a(x) and R1b, which became prevalent once they arrived in Europe.

### Geographic Y-chromosome sub-structure within the Iberian Peninsula

**Fig. 4:**
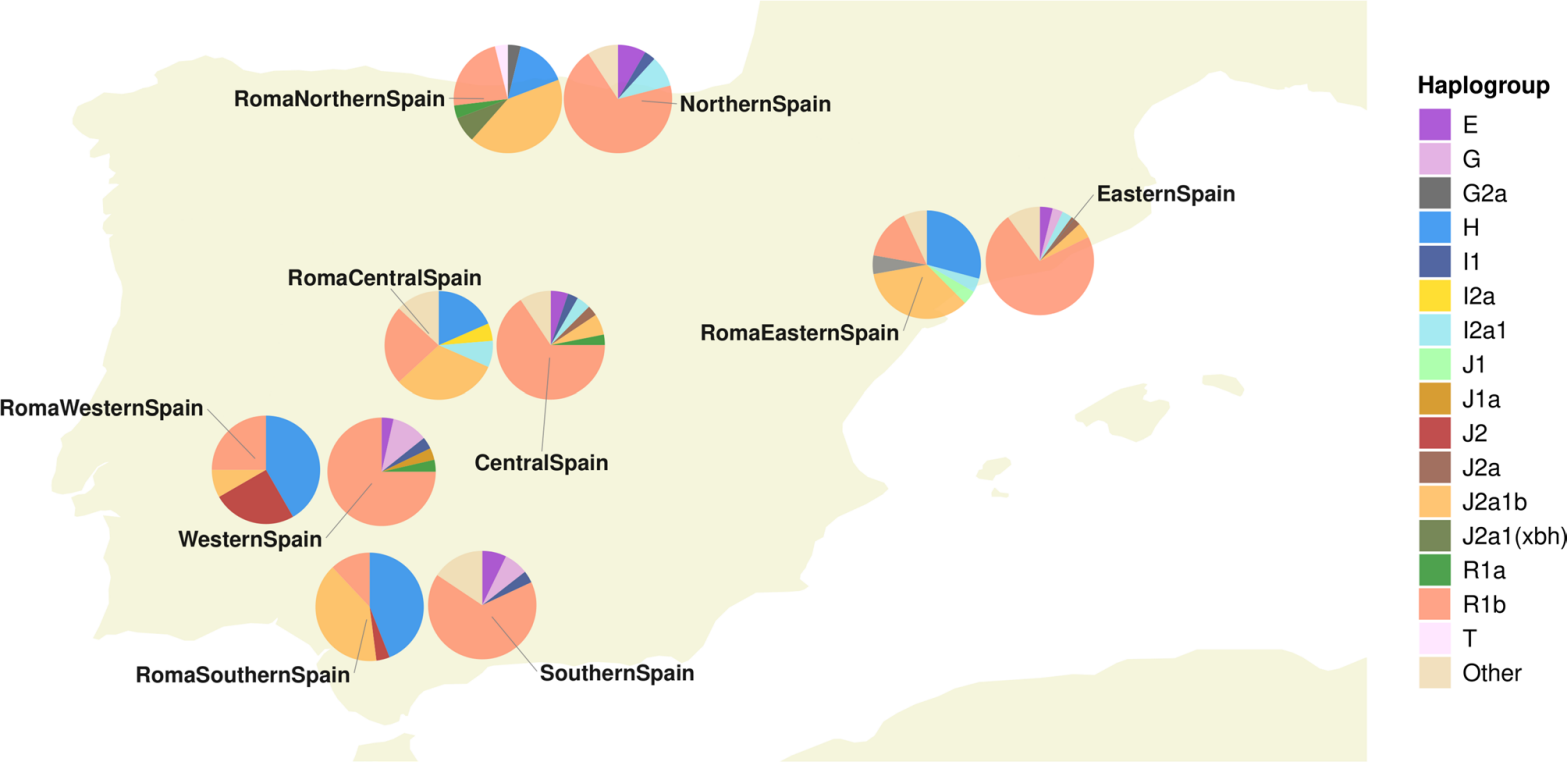
Geographic distribution of Y-chromosome haplogroups in Spanish Roma and non-Roma populations. Pie charts show the frequencies of inferred haplogroups in each population. Haplogroups with frequencies below 3% are labelled as “Other” in the legend

To examine the Spanish Roma at a finer geographic scale and compare genetic variation across Spanish regions, we measured and compared haplogroup composition and haplotype diversity within regional groups of Spanish Roma and non-Roma individuals (Table S6 and S7). The Spanish Roma groups exhibit lower haplotype diversity than the Spanish non-Roma (Table S8), likely due to smaller sample sizes and reduced genetic variability, which may result from serial population bottlenecks within the Roma population. Comparisons of haplogroup distributions between Spanish Roma and non-Roma populations reveal substantial differences, with no clear regional pattern linking Roma groups to geographically close non-Roma populations. Notable differences include the exclusive presence of haplogroup H and a higher prevalence of J2a1b in Roma groups, and the dominance of R1b in all Spanish non-Roma groups. The haplogroup distributions among Roma groups across different regions of Spain also show regional variation, which may reflect the impacts of differential admixture, isolation, and genetic drift (Table S9, Fig. 4).

Haplogroup H, associated with South Asian ancestry, is most frequent in Roma from Southern and Western Spain, with its frequency decreasing in Central and Northern Spain (Fig. 4, Table S9). In contrast, J2a1b, linked to West Asian ancestry, is more common in Northern and Eastern Spain (Fig. 4, Table S9). R1b, the dominant haplogroup in non-Roma Europeans, shows regional variability, with higher frequencies in Northern and Central Spain, suggesting increased gene flow with non-Roma populations in these areas (Fig. 4, Table S9). Additionally, less frequent haplogroups, such as I2 and its sub-haplogroups, exhibit regional patterns, appearing exclusively in Central and Eastern Spain (Fig. 4, Table S9). Together, these findings indicate diverse genetic influences and highlight regional substructure within Roma populations across Spain.

We next assessed the extent of genetic differentiation within the Spanish Roma using AMOVA to better understand internal heterogeneity and potential regional substructure (Table S10). Comparisons between the Y-chromosome composition of Spanish Roma and non-Roma populations showed significant differentiation, with 11.28% (*p* < 0.05) of the variation observed between these groups. When analysing the Spanish Roma and Spanish non-Roma populations by region, we found that 19.77% of the genetic variation could be attributed to differences between these populations (*p* < 0.05) with no significant differences within groups (0.9%, *p* > 0.05). Further exploration of genetic variation within Spanish Roma and Spanish non-Roma populations revealed that regional differentiation was over six times higher among Spanish Roma (3%, *p* < 0.05) compared to Spanish non-Roma (0.48%, *p* < 0.05), highlighting greater internal heterogeneity within the Roma group.

### Estimation of migration rates in the Roma population

To better understand regional substructure within Spain and quantify shared gene flow between groups, we used Slatkin’s RST to calculate the migration rate (M) between Spanish regions (Table S11). Our analysis reveals that in all pairwise comparisons, the M values exceed 1, indicating the presence of gene flow. Notably, M values among Spanish non-Roma regions are exceptionally high (M > 40), suggesting an absence of population substructure. In contrast, M values between Roma regions are generally around 1, indicating more limited levels of gene flow and supporting the presence of regional substructure, consistent with our AMOVA results. Furthermore, moderate gene flow (M > 1 and < 2) was observed between Roma and Spanish non-Roma regions, with no distinctive pattern among regions. However, this demonstrates the existence of at least some level of gene flow between the Roma and Spanish non-Roma across all regions.

To explore migration patterns within and into the Iberian Peninsula, we analysed five distinct migration models, focusing on the Iberian Roma (Spanish Roma divided by geographic regions and Portuguese Roma) and their genetic structure (Table S12 A). The model with the highest likelihood indicates that genetic differentiation among regions within the Iberian Roma is best explained by divergence from a common ancestor, likely driven by historical isolation and genetic drift, with minimal recent gene flow between regions (Figure S9). The second most likely model assumes divergence with ongoing east-to-west migration, suggesting that the Spanish Roma populations originated from eastern ancestors, with continuous gene flow between eastern and western Spanish Roma groups in a west-to-east cline (Table S12 A).

To trace the movements of the Roma from southwestern Europe into the Iberian Peninsula, we broadened the scope of our analysis, testing migration models across Roma populations from southern and central Europe to the Iberian Peninsula (Table S12 B). The highest likelihood model, which assumes migration from East to West—spanning Greece, Romania, Slovakia, and Spain—indicates that the genetic structure of the Spanish Roma aligns with a stepwise westward migration process through these regions (Figure S10). This pattern suggests sequential movement with genetic differentiation likely occurring at each stage, consistent with historical routes of Roma dispersal across Europe, and limited gene flow between populations in different regions during this westward migration. The second most likely model assumes divergence from a common ancestor along a west-to-east cline, indicating that all European Roma trace their origins to eastern ancestors, with continuous gene flow from eastern to western Roma groups.

## Discussion

The paternal genetic diversity observed within the Spanish Roma can be explained by demographic events including bottlenecks, founder effects, and multiple episodes of admixture with populations encountered throughout their diaspora, as well as following their settlement in the Iberian Peninsula. The Spanish Roma exhibit a distinctive paternal genetic profile with lower frequencies of South Asian haplogroups and a higher representation of Western European lineages, in contrast to Roma groups outside the Iberian Peninsula. This profile is characterised by a predominance of haplogroups J2a1b (34.1%) and H (27.7%), associated with West Asian and South Asian origins, respectively [64, 65]. For comparison, the frequency of haplogroup H in other European Roma ranges from a minimum of 28.6% in Ukrainian Roma to 67.2% in Greek Roma, possibly showing lower admixture with non-Roma. In the case of J2a1b, apart from Romanian Roma (30.7%), frequencies in other groups range from 19.8% in Portuguese Roma to 5.1% in Serbian Roma, suggesting that the increased frequency in Spanish Roma may result from genetic drift and endogamous practices, as observed with other haplogroups in previous studies on other Roma groups [66–68]. It is important to note that endogamy, combined with the practice of patrilocal residence observed both historically and in modern times among Spanish Roma [69, 70]—where women traditionally moved to their husband’s community—has likely influenced haplogroup frequencies in both paternal and maternal lineages. Overall, the frequency of these haplogroups in the Roma is consistent with their South Asian origin and subsequent migration through West Asia during their diaspora. Haplogroup R1b, commonly associated with Central and Western European populations [71], is found at a relatively high frequency in Spanish Roma (18.5%) compared to non-Iberian Roma groups in our dataset, where it remains below 8%. This haplogroup is particularly prevalent in Spanish (68%) and Basque (88%) populations, as estimated from data combined from earlier research [33, 72–74]. Taken together, this suggests substantial gene flow from Iberian populations into the Roma following their arrival in the region. This pattern of admixture with non-Roma populations in Iberia aligns with findings from autosomal DNA studies [21, 37] and likely reflects the history of forced assimilation occurred within the peninsula over several centuries [16, 23, 75].

Therefore, Roma populations likely arrived in Europe with existing diversity within haplogroups H and J2a1b, later incorporating additional lineages such as R1b and I2a(x), which are also prevalent in Europe [76], through admixture with local European populations. This process is reflected in the median-joining networks and MDS analyses, where haplogroups H and J2a1b show tight clustering and higher frequencies with minimal differentiation from other European Roma. In contrast, the R1b and I2a(x) haplogroups exhibit greater diversity and dispersion but occur at lower frequency in Roma compared to non-Roma populations, likely indicating multiple admixture events with European populations during their migration through Europe. Collectively these results align with historical accounts of the Roma’s migration routes [2, 3] and are consistent with previous genetic studies on mtDNA [32], Y-chromosome [33, 68], and whole-genome array data [21] in Spanish Roma.

To explore alternative routes of the diaspora into Iberia, we examined genetic relationships with North African and Jewish groups. Our analyses reveal no significant North African influence on the Spanish Roma paternal gene pool, despite its presence in the non-Roma Spanish population [77], where haplogroup E-M81, associated with Amazigh ancestry [78–80], accounts for around 1%. This contrasts with the Portuguese Roma, where 3.2% carry E haplogroups of African origin [22]. In comparison, predictions for the Portuguese non-Roma, based on combined data from multiple studies [81, 82], indicate that 12% carry the E1b1b haplogroup. However, due to the lack of Y-SNP data, we cannot confirm how many of these haplotypes are specifically of North African origin. The presence of E haplogroups in the Portuguese Roma, but not in the Spanish Roma, suggests that North African haplogroups entered the Roma gene pool via non-Roma Iberian populations—who had previously admixed with North African groups—rather than through direct contact between Roma and North Africans before their arrival in Iberia. Besides, as 2–3% North African ancestry was observed in the autosomal DNA of Iberian Roma [21], the absence of E haplogroups in our Spanish Roma sample may reflect random variation and the haplogroup’s low frequency in the population. These findings corroborate mtDNA studies [32], which also found no evidence of North African gene flow into the Spanish Roma. Additionally, we found no genetic link between Spanish Roma and Jewish populations, as paternal lineages commonly associated with Jewish ancestry were absent in the Spanish Roma and rarely present in other Roma groups. For example, J1-P58, which is predominant in several Jewish groups [83], was observed only twice in the Spanish non-Roma and in Bulgarian Roma, while E-M34 was detected in Serbian Roma at a frequency of 3.8%. These results suggest no evidence of admixture between Spanish Roma and Jewish populations, consistent with prior autosomal DNA findings [21].

Increased genetic drift, resulting from bottlenecks, founder events, and periods of isolation, has contributed to the preservation at high frequencies of specific founder lineages—defined by their presence in Roma and absence in non-Roma European populations [33, 36]— within the Roma population [84]. In this context, previous research has identified both maternal and paternal founder lineages within the Roma population [32, 33, 36]. These lineages include South Asian haplogroups (such as H-52 and H-M82) and Western European haplogroups (I-P259, J-M92, and J-M67), which has been explained as a result of a single Roma origin from North-Western India, with admixture and bottlenecks during their diaspora through Middle East and Europe. Despite the limited resolution of our STR-based data, we identified the presence of some paternal founder lineages such as H1a1 and J2a1b*. Beyond that, evidence from the median-joining network analysis shows that some newly-genotyped samples, particularly within haplogroups such as H and J2a1b, cluster with reference individuals carrying known founder lineages.

While mtDNA studies reported an 86% West-Eurasian and 14% South Asian maternal heritage in Iberian Roma [32], our results show a significantly higher South Asian contribution (30%) on the paternal side in the Spanish Roma. This discrepancy aligns with observations of sex-biased admixture in European Roma when analysing complete mtDNA and Y-chromosome sequences [35]. The migration of the proto-Roma appears to have been mostly male-driven, with South Asian paternal lineages preserved and maintained at higher frequencies in the population, while a large amount of West Eurasian maternal lineages was incorporated during their diaspora. Cultural customs, where non-Roma women are more likely to join the community through marriage with Roma men [85–87], likely explain, at least in part, the observed sex-biased ancestry.

We provide evidence of regional population structure within the Spanish Roma, as AMOVA analysis shows that genetic differentiation among geographic regions is six times greater than in the Spanish non-Roma. This is consistent with autosomal DNA findings [21] but contrasts with maternal DNA studies [32], which showed less pronounced inter-regional differentiation. This suggests that the preferential integration of non-Roma women has likely homogenised mtDNA across regions, while more restricted and region-specific paternal gene flow has led to the higher differentiation observed in Y-DNA and autosomal markers. Although Roma groups have experienced historical gene flow between regions, prolonged isolation during later periods—potentially linked to the transition from a nomadic to a settled lifestyle—may have contributed to the observed genetic substructure. This is supported by the migration rate analysis, which reveals significant but low M values between Roma populations across different Spanish regions—substantially lower than those observed between non-Roma Spanish from different regions. This may depend on the patrilocal residence patterns [69, 70], which could help explain the reduced paternal gene flow between regions.

Finally, we contribute to understanding Roma migrations in the Iberian Peninsula and Europe by applying coalescent methods and Bayesian demographic model inference. At the European scale, the model with the highest likelihood suggests a westward migration pattern, consistent with previous autosomal DNA findings [21, 37], which identified multiple migration waves from the Balkans and Southwestern Europe towards Iberia, as well as with historical sources [2, 3]. At the Iberian scale, the model with the highest likelihood indicates divergence from ancestral populations without ongoing migration. These findings suggest that the Roma in the Iberian Peninsula diverged early from their ancestral populations and subsequently experienced limited gene flow. This supports the presence of a genetic structure shaped by geographic distribution and highlights distinctive genetic patterns within Iberian Roma compared to broader European Roma populations.

## Conclusions

This study sheds light on the paternal genetic profile of Spanish Roma, highlighting the influence of different populations, demographic processes and cultural practices on their genetic structure. These findings contribute to the broader understanding of Roma migration patterns in Europe, offering valuable insights into the complex demographic history of Spanish Roma. While Y-STR analysis provides valuable insight into the paternal genetic structure of the Spanish Roma, its resolution is limited compared to whole Y-chromosome sequencing. Future studies should prioritise generating high-resolution paternal sequence data from a diverse set of European Roma populations to detect deep genealogical branches, identify new founder lineages, and capture a broader range of genetic diversity. Integrating this with autosomal, mtDNA and X-chromosome analyses will provide a more comprehensive view of Roma’s demographic history.

## Supplementary Information

Supplementary Material 1.

Supplementary Material 2.

## Data Availability

The newly generated raw Y-STR data are available in Supplementary Table S2.
