# Supplementary material for: Demographic insights into paternal genetic diversity and regional substructure in the Spanish Roma
Source: BMC Genomics. 2025 Nov 7;26:1012. doi: 10.1186/s12864-025-12210-8 (PMC12595825; doi:10.1186/s12864-025-12210-8)
Supplement: Supplementary file 1 — Supplementary Material 1. [file 12864_2025_12210_MOESM1_ESM.docx]

**Supplementary Figures**

*Demographic insights into paternal genetic diversity and regional substructure in the Spanish Roma*

*Giacomo F. Ena^1^, Aaron Giménez^2^, Annabel Carballo-Mesa^3^, Marcos Araújo Castro e Silva^1^, David Comas^1, *^*

*^1^ Institut de Biologia Evolutiva (CSIC-UPF), Universitat Pompeu Fabra, Departament de Medicina i Ciències de la Vida, Barcelona*

*^2^ Facultat de Sociologia, Universitat Autònoma de Barcelona, Barcelona, Spain*

*^3^ Facultat de Geografia i Història, Universitat de Barcelona, Barcelona, Spain*

*^*^Corresponding author*

*david.comas@upf.edu*


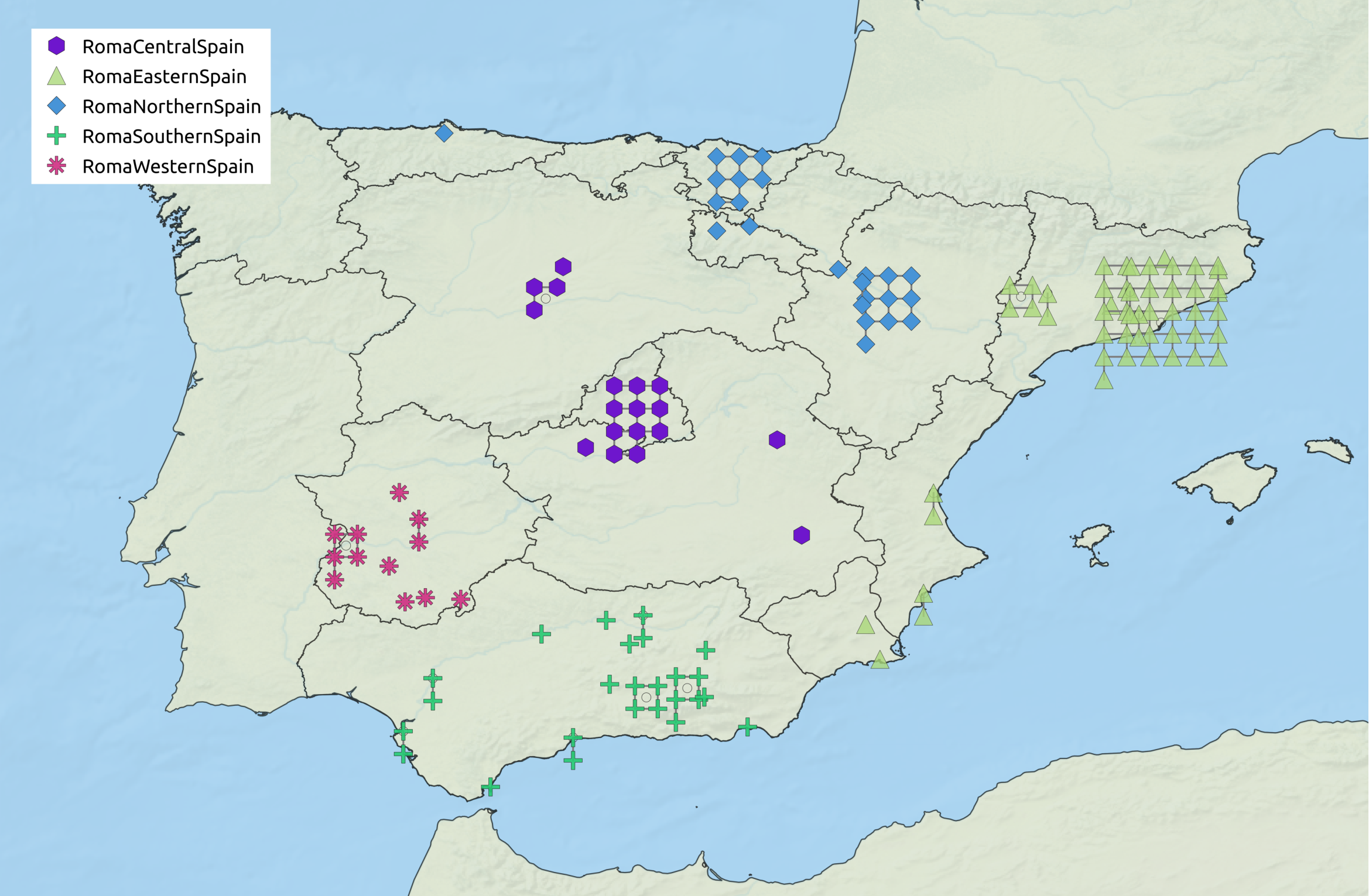


**Figure S1. Geographic distribution of Spanish Roma individuals throughout Spain.** Map created in QGIS software.


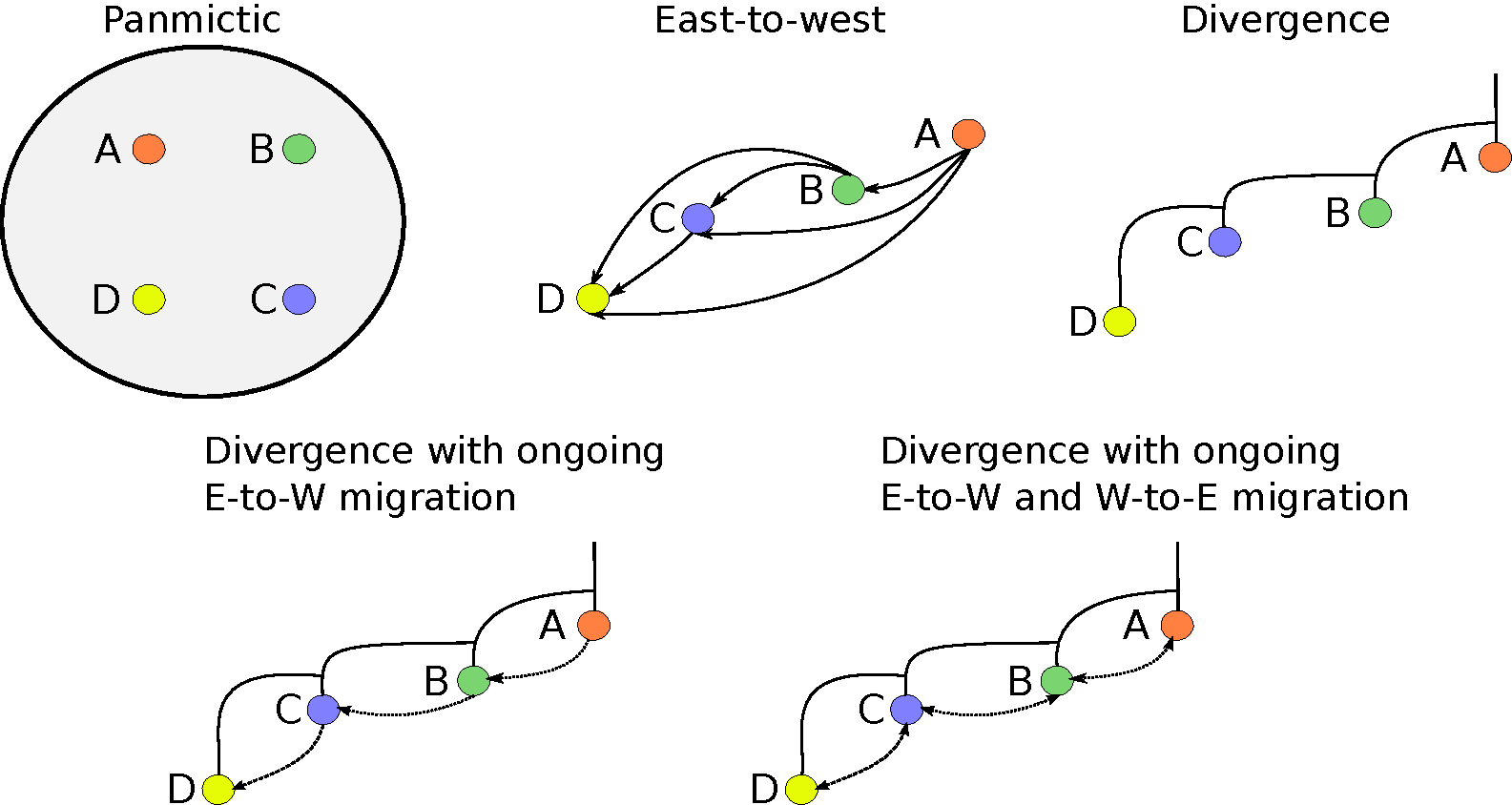


**Figure S2. Five migration model routes used to investigate the movements of Roma populations.**


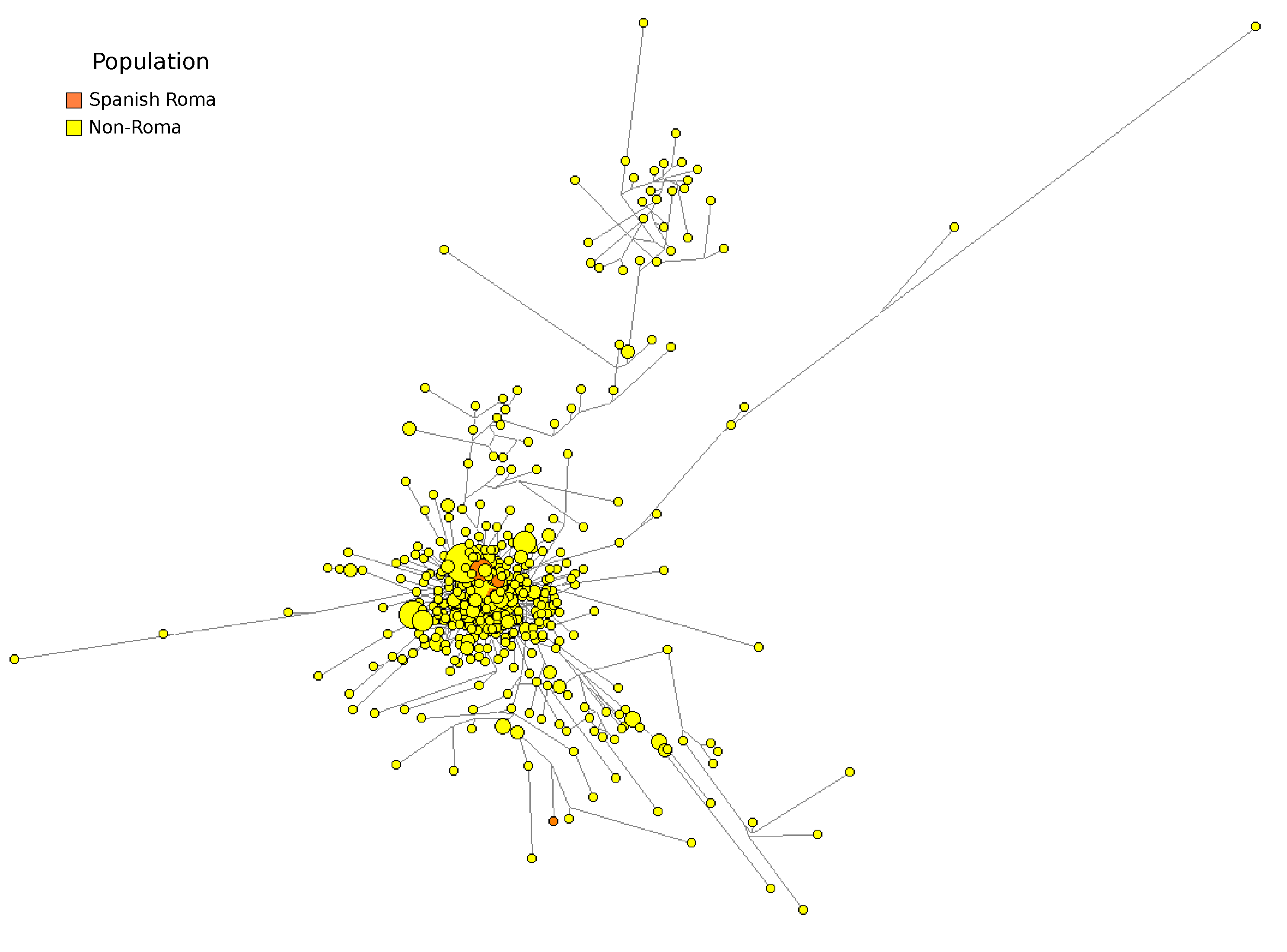
**Figure S3. Median-joining network of Y-STR haplotypes showing the distribution of predicted H haplogroups across Spanish Roma and all reference populations.** The network includes a random sample of up to 20 individuals per non-Roma groups.


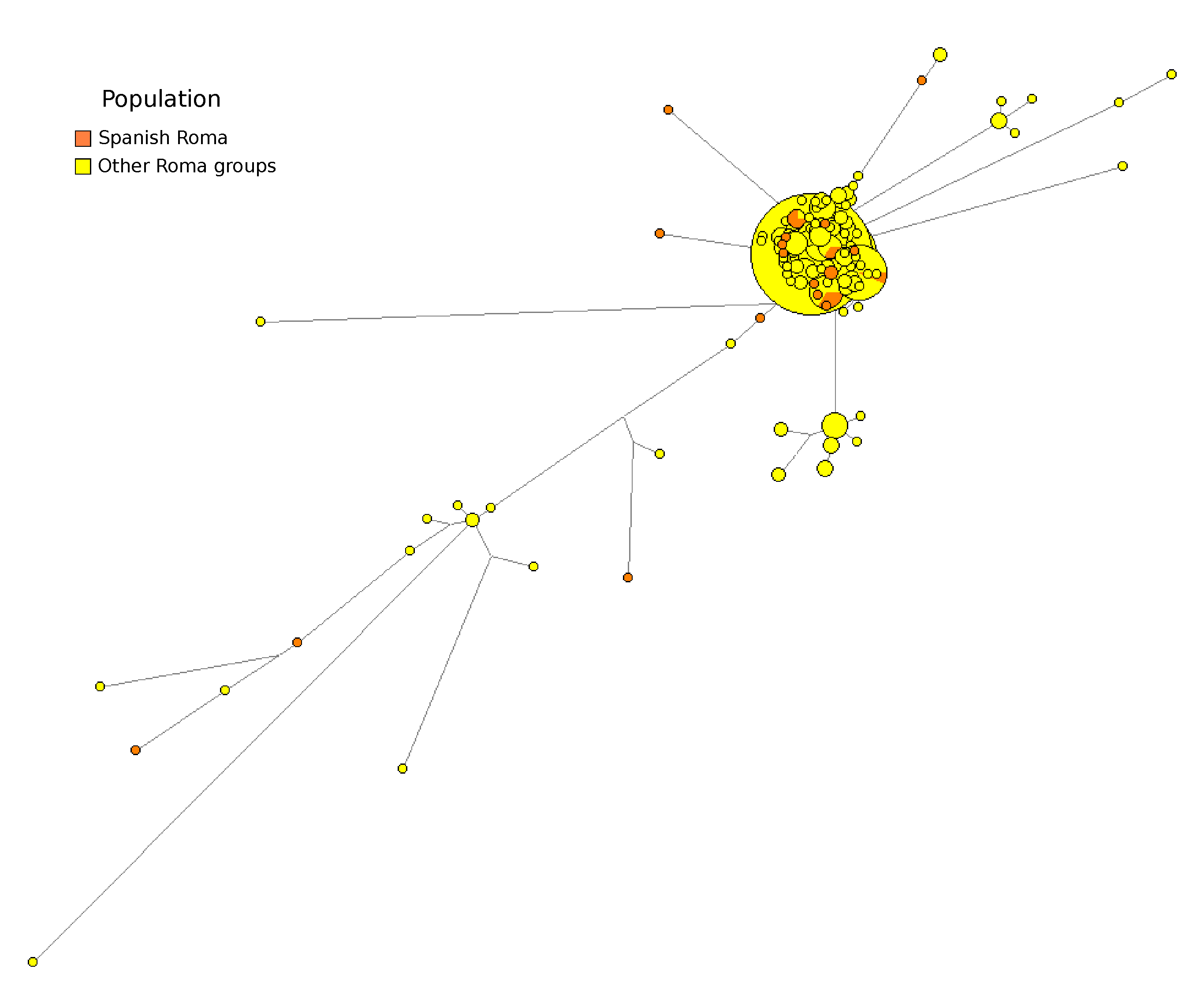


**Figure S4. Median-joining network of Y-STR haplotypes showing the distribution of predicted H haplogroups across Roma groups.**


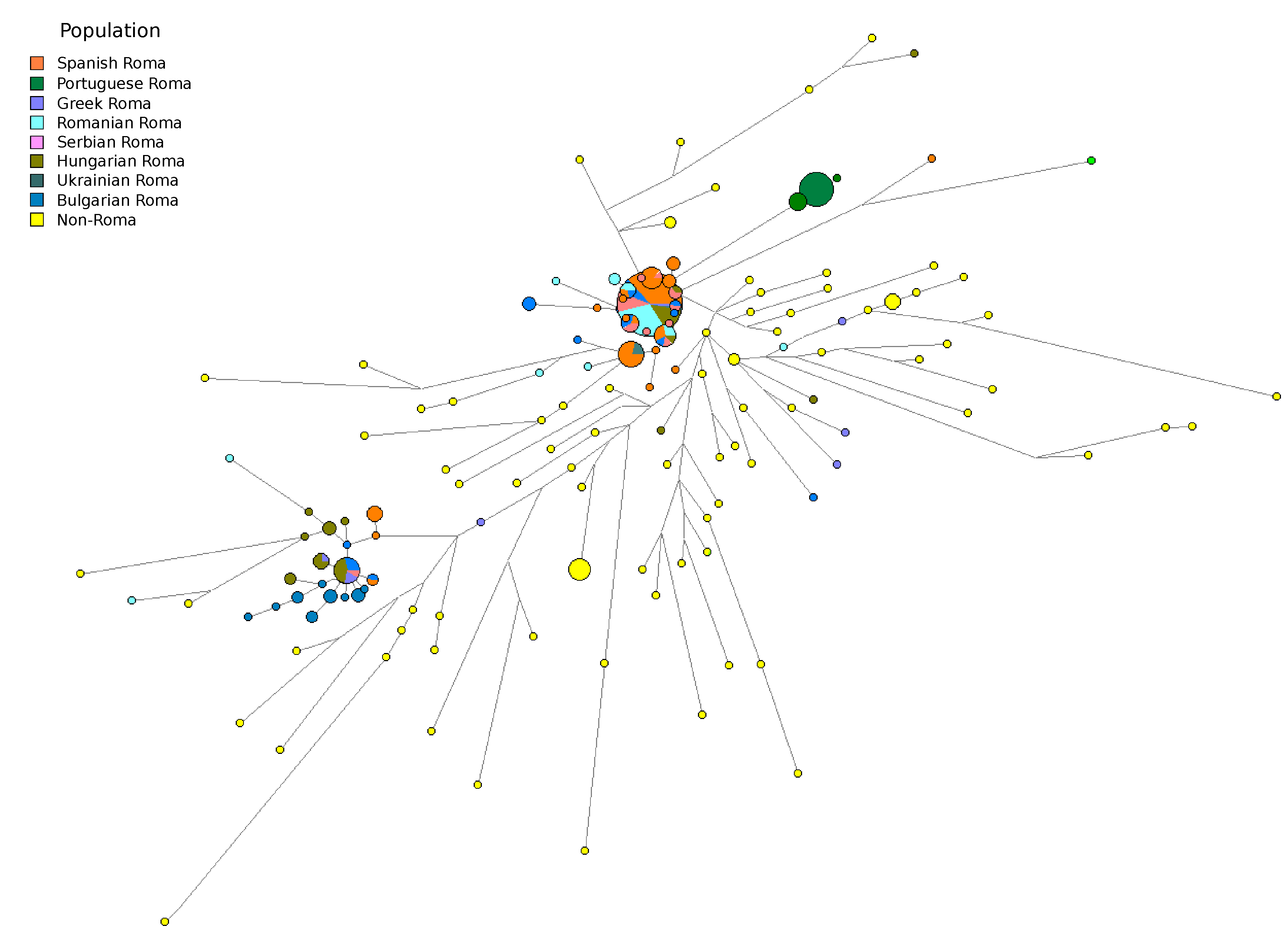


**Figure S5. Median-joining network of Y-STR haplotypes showing the distribution of predicted J2a1b haplogroups across all populations.**


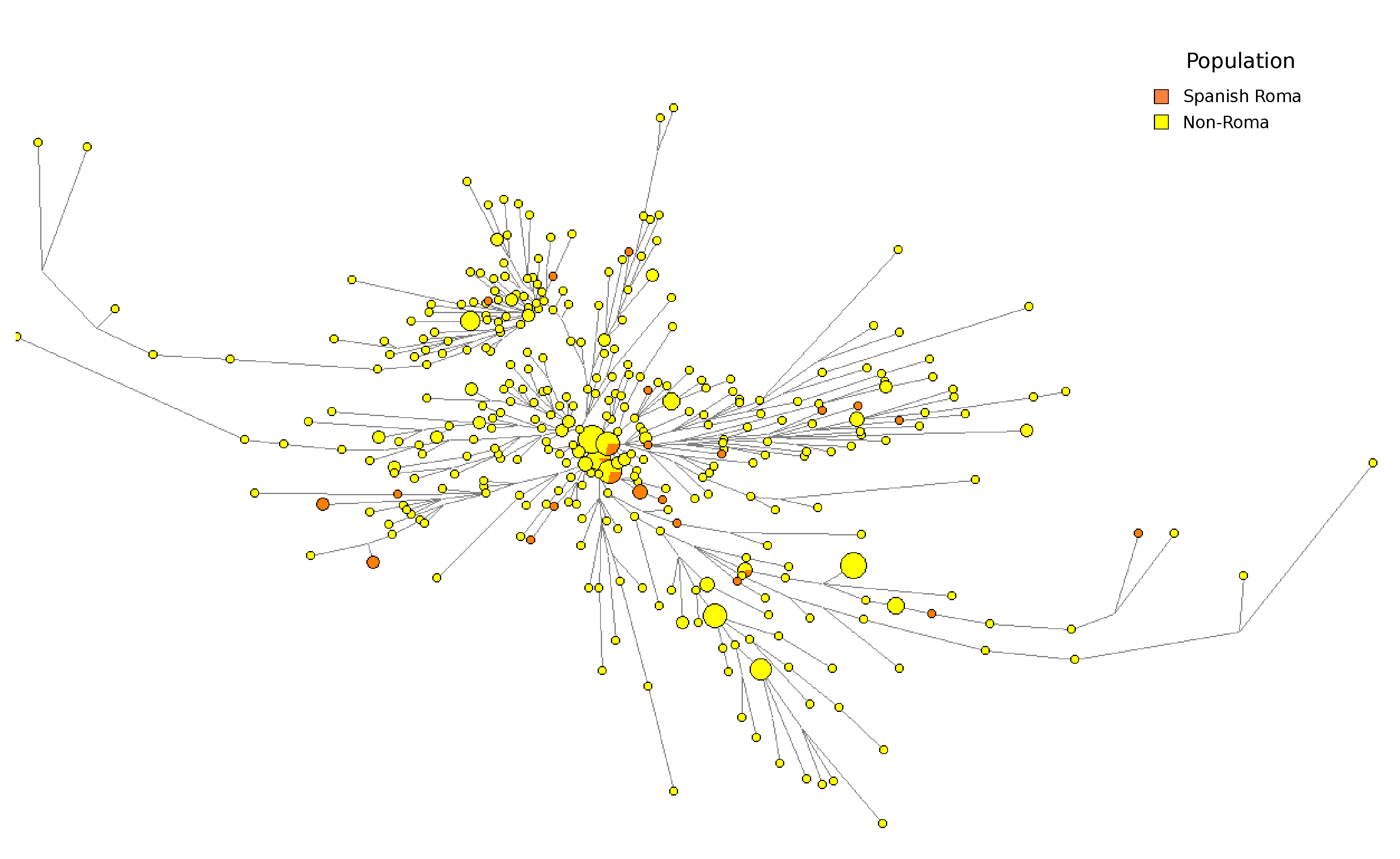
**Figure S6. Median-joining network of Y-STR haplotypes illustrating the distribution of predicted R1b haplogroups across Spanish Roma and all reference populations.** The network includes a random sample of up to 20 individuals per non-Roma groups.


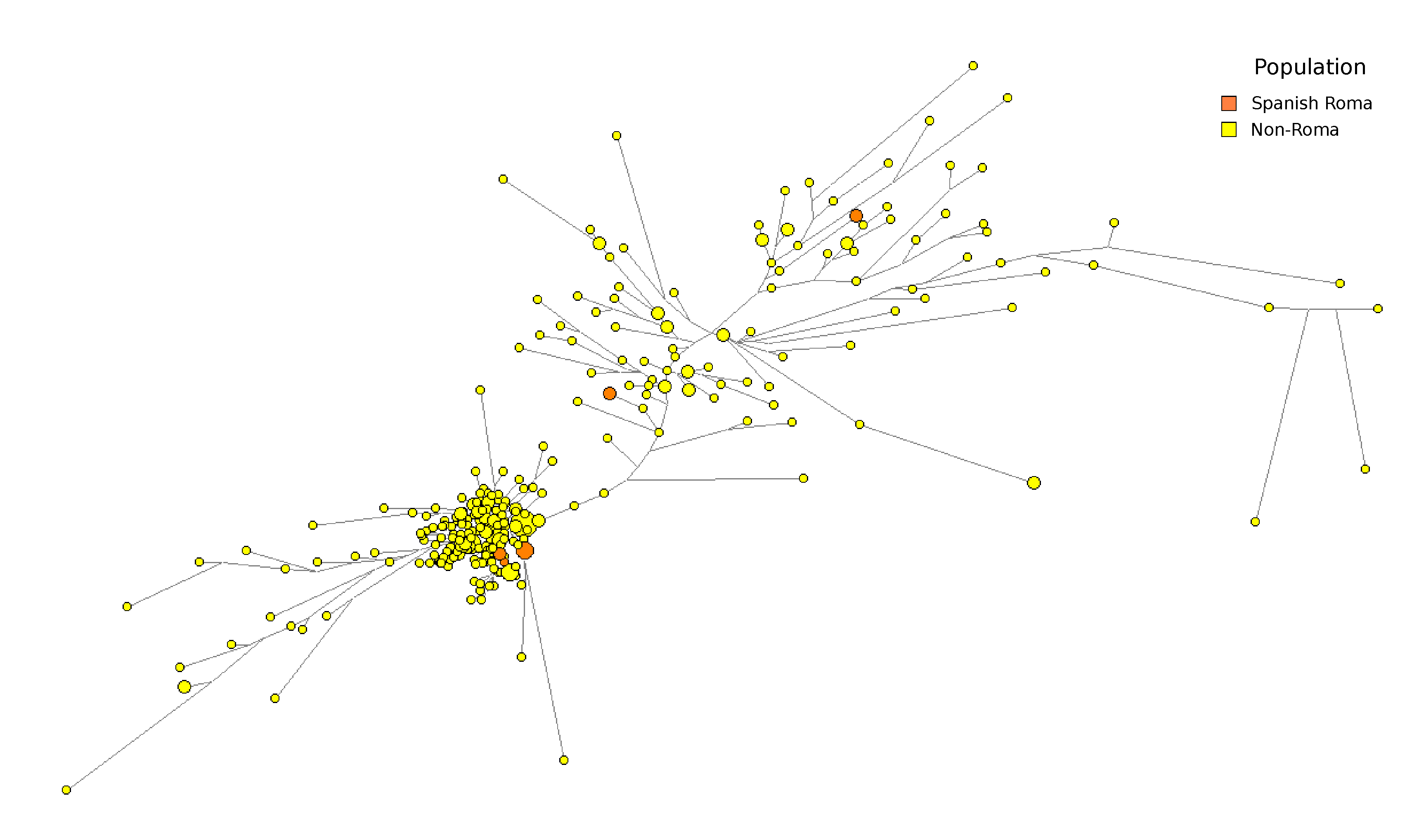
**Figure S7. Median-joining network of Y-STR haplotypes illustrating the distribution of predicted I2a(x) haplogroups across Spanish Roma and all reference populations.** The network includes a random sample of up to 20 individuals per non-Roma groups.


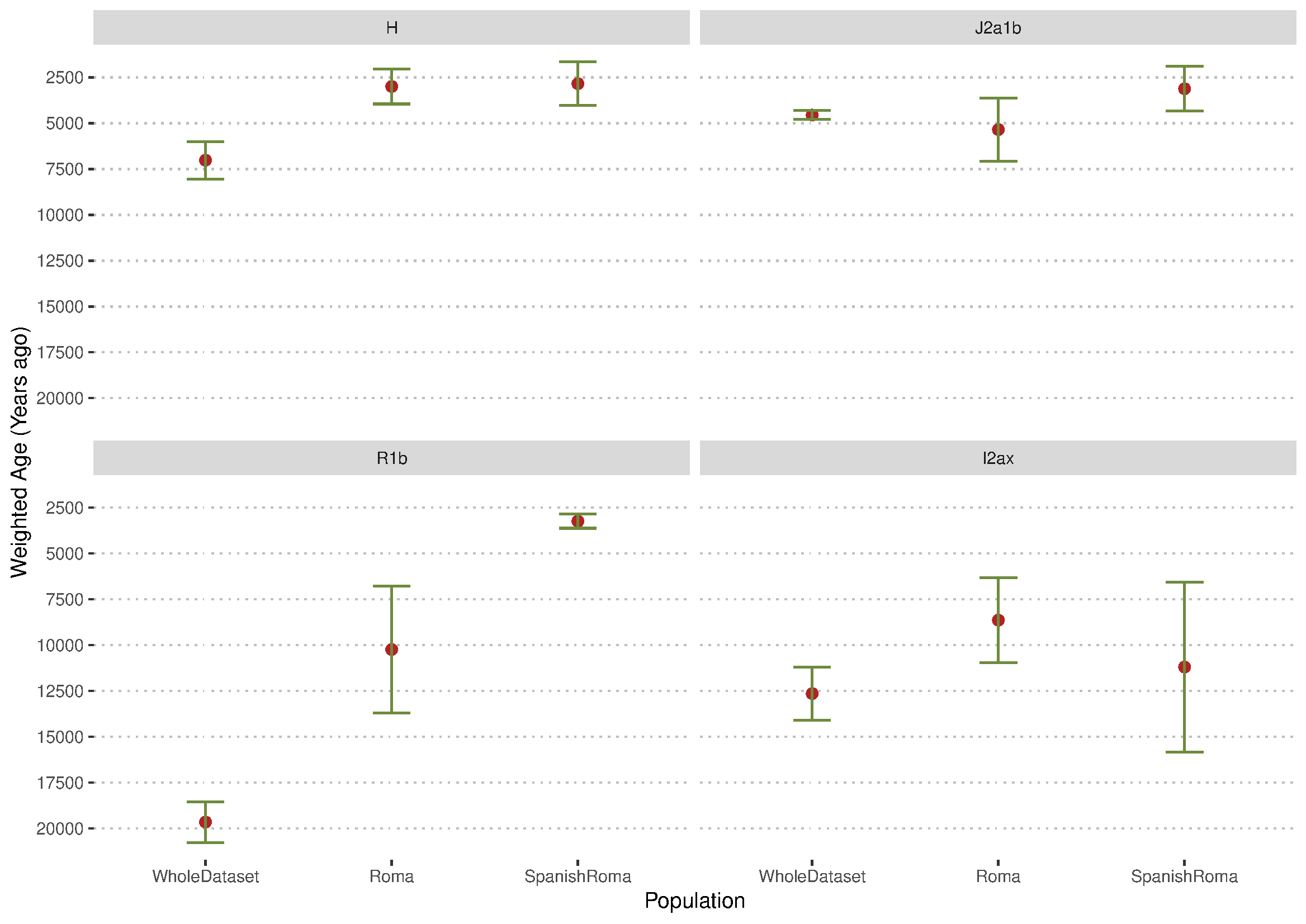
**Figure S8. Time to the most recent common ancestor (TMRCA) estimates for the four major haplogroups, calculated using the rho statistic and STR-based mutation rates, and evaluated at three hierarchical levels.**


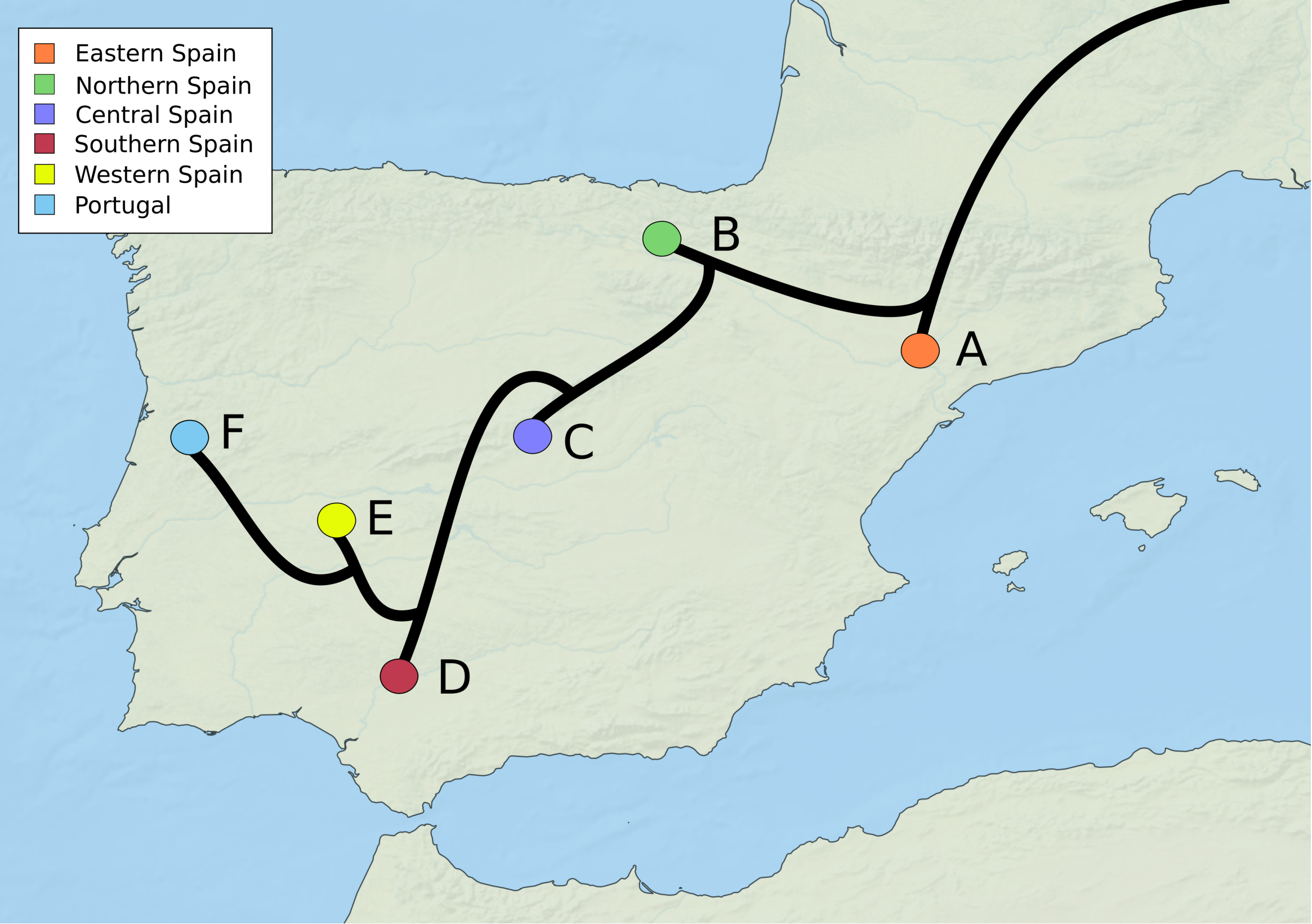
**Figure S9. The most dominant migration routes of Roma individuals from the six different regions of the Iberian Peninsula.** Map created in QGIS software.


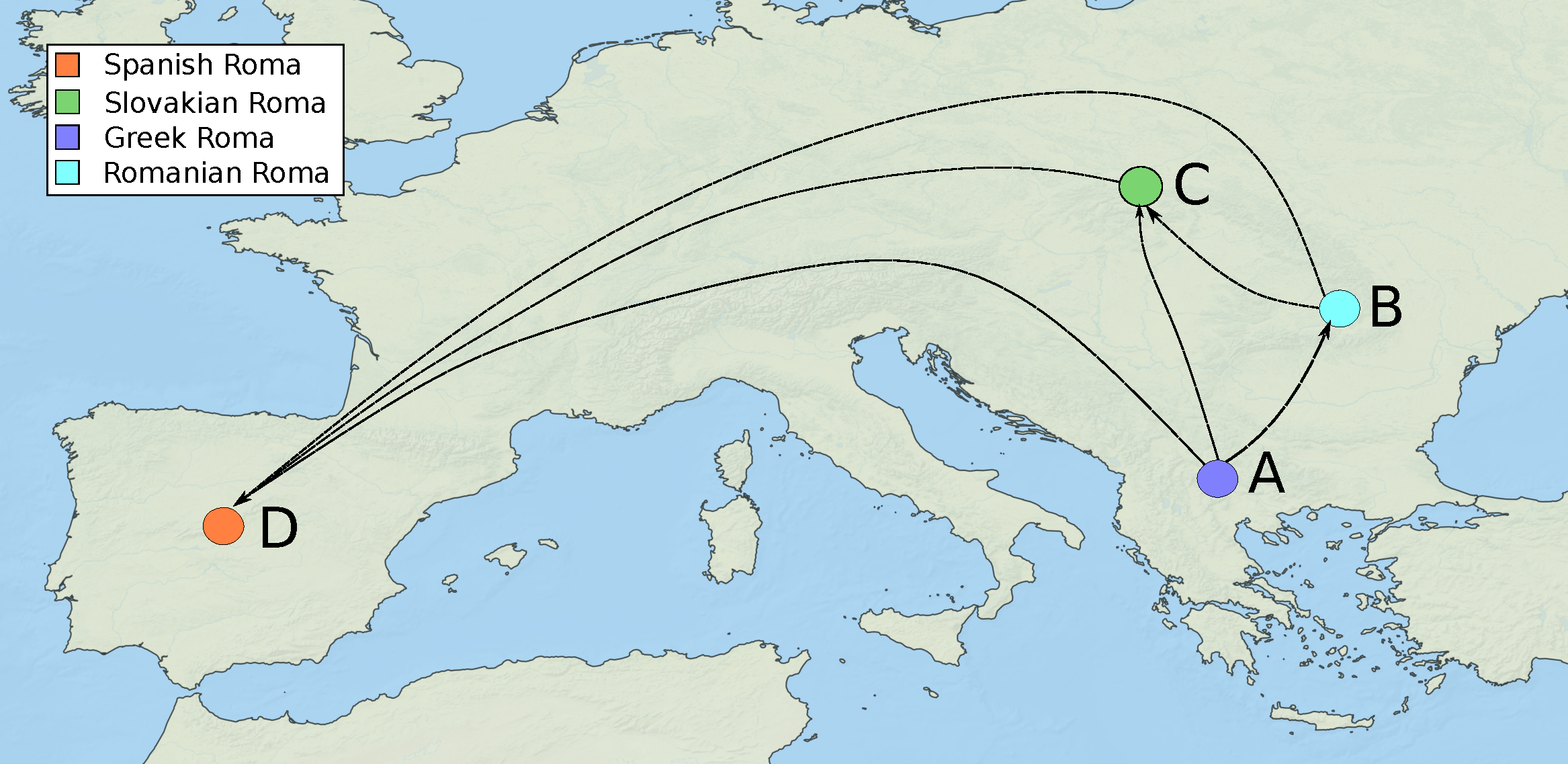
**Figure S10. The most dominant migration routes between Roma populations in the Iberian Peninsula, Slovakia, Romania, and Greece.** Map created in QGIS software.
